# Supplementary material for: Heat adaptation of phage T7 under an extended genetic code
Source: Virus Evol. 2021 Dec 1;7(2):veab100. doi: 10.1093/ve/veab100 (PMC8923235; doi:10.1093/ve/veab100)
Supplement: veab100_Supp [file veab100_supp.zip › Sequencing_Analysis.zip!/Sequencing_Analysis/A1/evidence/JC_SIDE_2_55.html]

BRESEQ :: Evidence


| New junction evidence | | | | | | | | | | | |
| --- | --- | --- | --- | --- | --- | --- | --- | --- | --- | --- | --- |
|  | | seq id | position | reads (cov) | reads (cov) | score | skew | freq | annotation | gene | product |
| \* | ? | NC\_001604.1 | = 13244 | 2141 (0.690) | 49 (0.020) | 39/582 | 27.3 | 4.5% | intergenic (–/–) | *–/–* | –/– |
|  | ? | NC\_001604.1 | 14067 = | 6 (0.000) |  |  |  |  | intergenic (–/–) | *–/–* | –/– |
| Rejected: Coverage evenness skew score above cutoff. | | | | | | | | | | | |
| Rejected: Frequency below/above cutoff threshold. | | | | | | | | | | | |

`TCATGAAGCTGGTCGCGTCAATGAAGCTATGGCTATCCTCGACAAGATTGATAACTAAGAGTGGTATCCTCAAGGTCGCCAAAGTGGTGGCCTTCATGAATACTATTCGACTCACTATAGGAGATATTACCATGCGTGACCCTAAAGTTATCCAAGCAGAAATCGCTAAACTGGAAGCTGAACTGGAGGACGTTAAGTACCATGAAGCTAAGACTCGCTCCGCTGTTCACATCTTGAAGAACTTAGGCTGGACTTGGACAAGACAGACTGGCTGGAAGAAACCAGAAGTTACCAAGCTGAGTCATAAGGTGTTCGATAAGGACACTATGACCCACATCAAGGCTGGTGATTGGGTTAAGGTTGACATGGGAGTTGTTGGTGGATACGGCTACGTCCGCTCAGTTAGTGGCAAATATGCACAAGTGTCATACATCACAGGTGTTACTCCACGCGGTGCAATCGTTGCCGATAAGACCAACATGATTCACACAGGTTTCTTGACAGTTGTTTCATATGAAGAGATTGTTAAGTCACGATAATCAATAGGAGAAATCAATATGA  >  NC_001604.1/13796‑14356`  

                                                                                                                                                                                                                                                                               `|`                                                                                                                                                                                                                                                                                                   
`tCATGAAGCTGGTCGCGTCAATGAAGCTATGGCTATCCTCGACAAGATTGATAACTAAGAGTGGTATCCTCAAGGTCGCCAAAGTGGTGGCCTTCATGAATACTATTCGACTAACTATAGGCGATATTACCATGCGTGACACAAAAGCTATCCAAGCCGAAATCGCTAAACTGGAAGCTGAACTGGAGGACGTTAAGTACCATGAAGCAAAGAATCGCTCCGCTGTTCACATCTTGAAGAAAGTAGGCTGGACTTGGACAAGACAGACTGGCTGgaagcagactctaagacacatctcaga                                                                                                                                                                                                                                                                      >  1:130826/1‑278 (MQ=255)`  
                      `aaaGCTATGGCTATCCTCGACAAGATTGATAACTAAGAGTCGTATCCTTCATGTCGCCAAAGTGGTGGCTTTCATGAATACTAGTCGACTCACTATAGGAGATATTACCATGCGTGACCCTAAAGTTATCCAAGCAGAAAACGCTAAACTGGAAGCTGAACTGGAGGACGTTAAGTACCATGAAGCTAAGACTCGCTCCGCTGTTCACGTCTTGAAGAACTTAGGCTGGACTTGGACAAGACAGACTGGCTGGAAGAAACCAGAAGTTACCAAGCGGAGTCATAAGGTGTTCGATAAGGac                                                                                                                                                                                                                                                <  1:293444/300‑1 (MQ=255)`  
                                                                                                            `gACTCACTATAGGAGATATTACCATGCGTGACCCTAAAGTTATCCAAGCAGAAATCGCTAAACGGGAAGCCGAACTGGAGGACGTTAAGTACCATGAAGCTAAGACTCGCTCCGCTGTTCACATCTTGAAGAACTTAGGCTGGACTGGGACAAGACAGACTGGCTGGAAGAAACCAGAAGTTACCAAGATGAGTCATAAGGTGTTCGATAAGGACACTATGACCCACATCAAGGCTGGTGAGTGGGTTAAGGTTGACATGGGAGTTGTTGGTGGATACGGCTACGTCCGCTCAGTTAGTgg                                                                                                                                                          <  1:433707/301‑1 (MQ=255)`  
                                                                                                                  `cTATAGGAGATATTACCATGCGTGACCCTAAAGTTATCCAAGCAGAAATCGCTAAACTGGAAGCTGAACTGGAGGACGTTAAGTACCATGAAGCTAAGACTCGCTCCGATGTTAACATCTTGAAGAACTTAGTCTGGACTTGGACAAGACAGACTGGCGGGAATAAACAAGAAGTTACCAAGCTGAGACATAAGGTGTTCGATAAGGACACTATGACCCACATCAAGGCTGGTGACTGGGTTAAGGTTGACTTGGGAGTTGTTGGTGGATACGGTTACGTCCGCTCAGTTAGTGGCaaaca                                                                                                                                                    >  1:314369/1‑299 (MQ=255)`  
                                                                                                                                                                                                                          `tCCGCTGTGAAAATCTTGAAGAACGTAGGCTGGACTGGGAAAAGACAGACTGGCGGGAAGAAACCAGAAGTTACCAAGCTGAGTCATAAGGGGTTCGATAAGGACACTAGGACCCACATCAAGGCTGGTGATTGGGTTAAGGTTGATATGGGAGTTGTTGGTGGATACGGCTACGTCCGCTCAGTTAGTGGTAAATATGCACAAGTGTCATACATCACAGGTGTTACTCCACGCGGTGCAATCGTTGCCGATAAGACCAACATGATTCACACAGGTTTCTTGACAGTTGTTTCATATGAag                                            <  1:50340/301‑1 (MQ=255)`  
                                                                                                                                                                                                                               `tGTTCACATCTTGAAGAACTTAGGCTGGACTTGGACAAGACAGACTGGCTGGAAGAAACCAGAAGTTACCAAGCTTAGTCATAAGGTGTTCGATAAGGACACTATGACCCACATCAAGGCTGGTGATTGGGTTAAGGTTGACATGGGAGTTGTTGGTGGATACGGCTACGTCCGCTCAGTTAGTGGCAAATATGCACAAGTGTCATACATCACAGGTGTTACTCCACGCGGTGCAATCGTTGCCGATAAGACCAACATGATTCACACAGGTTTCTTGACAGTTGTTTCATATGAAGAGAtt                                       <  1:88410/301‑1 (MQ=255)`  
                                                                                                                                                                                                                                            `tggatgaagagtcacactcagagtcaacagacTGGCTGGAAGAAACCATAAGTTACCAAGCTTAGTCATAAGGTGTTCGATAAGGACACTATGACCCACATCAAGGCTGGTGATTGGGTTAAGGTTGACATGGGAGTTGTTGGTGGATACTGCTACGTCCGCTCAGTTAGTGGCAAATATGCACAAGTGTCATACATCACAGGTGTTACTCCACGCGGTGCAATCGTTGCCGATAAGACCAACATGATTCACACAGGTTTCTTGACAGTTGTTTCATATGAAGAGATTGTTAAGTCACGAt                          <  1:148349/275‑1 (MQ=255)`  
                                                                                                                                                                                                                                            `gggaagaagagtcacactcagagtcaaaagaaTGGCTGGAAGAAACCAGAAGTTACCAAGCTGAGTCATAAGGTGTTCGATAAGGACACTATGACCCACATCAAGGCTGGTGATTGGGTTAAGGTTGACATGGGAGTTGTTGGTGGATACTGCTACGTCCGCTCAGTTAGTGGCAAATATGCACAAGTGTCATACATCACAGGTGTTACTCCACGCGGTGCAATCGTTGCCGATAAGACCAACATGATTCACACAGGTTTCTTGACAGTTGTTTCATATGAAGAGATTGTTAAGTCACGAt                          <  1:148370/273‑1 (MQ=255)`  
                                                                                                                                                                                                                                             `ggaagaagagtcacactcagagtcaacagacTGGCTGGAAGAAACCAGAAGTTACCAAGCTGAGTCATAAGGTGTTCGATAAGGACACTATGACCCACATCAAGGCTGGTGATTGGGTTAAGGTTGACATGGGAGTTGTTGGTGGATACGGCTACGTCCGCTCAGTTAGTGGCAAATATGCACAAGTGTCATACATCACAGGTGTTACTCCACGCGGTGCAATCGTTGCCGATAAGACCAACATGATTCACACAGGTTTCTTGACAGTTGTTTCATATGAAGAGATTGTTAAGTAACGATa                         <  1:447558/276‑1 (MQ=255)`  
                                                                                                                                                                                                                                              `gaagaagagtcacactcagagtcaacagacTGGCTGGAAGAAACCAGAAGTTACCAAGCTGAGTCATAAGGTGTTCGATAAGGACACTATGACCCACATCAAGGCTGGTGATTGGGTTAAGGTTGACATGGGAGTTGTTGGTGGATACGGCTACGTCCGCTCAGTTAGTGGCAAATATGCACAAGTGTCATACATCACAGGTGTTACTCCACGCGGTGCAATCGTTGCCGATAAGACAAACATGATTCACACAGGTTTCTTGACAGTTGTTTCATATGACAAGATTGTTAAGTCACGATaa                        >  1:293159/25‑301 (MQ=255)`  
                                                                                                                                                                                                                                                 `gaagagtcacactcagagtcaacagacTGGCTGTAAGAAACCAGAAGTTACCAAGCTGAGTCATAAGGTGTTCGATAAGGACACTATGAACCACATCAAGGATGGTGATTGGGTGAAGGTTGAAATGGGAGTTGTTGGTGGATACGGCTACGTCCGCTCAGTTAGTGGCAAATATGCACAAGTGTCATAAATCGCAGGTGTTAATCCACGCGGTGCAATCGTTGCCGATAAGACCAACATGATTCCAAAAGGTTTCTTGACAGTTGGTTCATATAAAGAGATTGTTAAGTCAAGATAAGCa                     >  1:346770/22‑301 (MQ=255)`  
                                                                                                                                                                                                                                                   `aagacagcactcagagtaaacagacTGGCTGGAAGAAACCAGAAGTTACCAAGCTGAGTCATAAGGTGTTCGATAAGGACACTATGACCCACATCAAGGCTGGTGATTGGGTTAAGGTTGACATGGGAGTTGTTGGTGGATACGGCTACGTCCGCTCAGTTAGTGGCAAATATGCACAAGTGTCATACATCACAGGTGTTACTCCACGCGGTGCAATCGTTGCCGATAAGACCAACATGATTCACACAGGTTTCTTGACAGTTGTTTCATATGAAGAGATTGTTAAGTCACGATAATCAAt                   <  1:407804/282‑1 (MQ=255)`  
                                                                                                                                                                                                                                                    `gagtcatactcagagtcaacagacTGGCTGGAAGAAACCAGAAGTTACCAAGCTGAGTCATAAGGTGTTCGATAAGGACACTATGACCCACATCAAGGCTGGTGATTGGGTTAAGGTTGACATGGAAGTTGTTGGTGGATACGGCTACGTCCGCTCAGTTAGTGGCAAATATGCACAAGTGTCATACATCACAGGTGTTACTCCACGCGGTGCAATCGTTGCCGATAAGACCAACATGATTCACACAGGTTTCTTGACAGTTGTTTCATATGAAGAGATTGTTAAGTCACGATAATCACTa                  >  1:435750/19‑301 (MQ=255)`  
                                                                                                                                                                                                                                                        `cacactaagagtcaacggacTGGATGGAAGAAACCAAAAGTTACCAAGTTGATTCATAAGGTGTTCGATAAGGACACTATGACCAACATCAAGGCTGGTGATTGGGTTAAGGTTGACATGGGAGTTGTTGTTGGATACGGCTACTCCTGCTCATTTAGTGGCAAATATGCACAAGTGTCATACATCACAGGTGTTACTCCACGCGGTGCAATCGTTGCCGATAAGACCAACATGATTCACACAGGTTTCTTGACAGTTGTTTCATATGAAGAGATTGTTAAGTCACGATAATCAATAGgag              <  1:302816/284‑1 (MQ=255)`  
                                                                                                                                                                                                                                                                 `agtcaacagacTGGCTGGAAGAAACCAGAAGTTACCAAGCTGAGTCATAAGGTGTTCGATAAGGACACTATGACCCACATCAAGGCTGGTGATTGGGTTAAGGTTGACATGGGAGTTGTTGGTGGATACGGCTACGTCCGCTCAGTTAGTGGCAAATATGCACAAGTGTCATACATCACAGGTGTTACTCCACGCGGTGCAATCGTTGCCGATAAGACCAACATGATTCACACAGGTTTCTTGACAGTTGTTTCATATGAAGAGATTGTTAAGTCACGATAATCAATAGgagctgtctctt     >  1:60962/6‑292 (MQ=255)`  
                                                                                                                                                                                                                                                                    `caacagacTGGCTTGAAGAAACCAGAAGTTACCAAGCTGAGTCATAAGGTGTTCGATAAGGACACTATGACCCACATCAAGGCTGGTGATTGGGTTAAGGTTGACATGGGAGTTGTTGGTGGATACGGCTACGTCCGCTCAGTTAGTGGCAAATATGCACAAGTGTCATACATCACAGGTGTTACTCCACGCAGTGCAATCGTTGCCGATAAGACCAACATGATTCACACAGGTTTCTTGACAGTTGTTTCATATGAAGAGATTGTTAAGTCACGATAATCAAtctgtctcttatacacat  >  1:24584/3‑284 (MQ=255)`  

                                                                                                                                                                                                                                                                               `|`                                                                                                                                                                                                                                                                                                   
`TCATGAAGCTGGTCGCGTCAATGAAGCTATGGCTATCCTCGACAAGATTGATAACTAAGAGTGGTATCCTCAAGGTCGCCAAAGTGGTGGCCTTCATGAATACTATTCGACTCACTATAGGAGATATTACCATGCGTGACCCTAAAGTTATCCAAGCAGAAATCGCTAAACTGGAAGCTGAACTGGAGGACGTTAAGTACCATGAAGCTAAGACTCGCTCCGCTGTTCACATCTTGAAGAACTTAGGCTGGACTTGGACAAGACAGACTGGCTGGAAGAAACCAGAAGTTACCAAGCTGAGTCATAAGGTGTTCGATAAGGACACTATGACCCACATCAAGGCTGGTGATTGGGTTAAGGTTGACATGGGAGTTGTTGGTGGATACGGCTACGTCCGCTCAGTTAGTGGCAAATATGCACAAGTGTCATACATCACAGGTGTTACTCCACGCGGTGCAATCGTTGCCGATAAGACCAACATGATTCACACAGGTTTCTTGACAGTTGTTTCATATGAAGAGATTGTTAAGTCACGATAATCAATAGGAGAAATCAATATGA  >  NC_001604.1/13796‑14356`

  

Alignment Legend

Aligned base mismatch/match (shaded by quality score): `ATCG/ATCG` `< 3 ≤` `ATCG/ATCG` `< 10 ≤` `ATCG/ATCG` `< 15 ≤` `ATCG/ATCG` `< 31 ≤` `ATCG/ATCG` `< 38 ≤` `ATCG/ATCG`

Unaligned base: `atcg`    Masked matching base: `atcg`    Alignment gap: `‑`    Deleted base: `‑`

Reads not counted as support for junction

read\_name Not counted due to insufficient overlap past the breakpoint.

read\_name Not counted due to not crossing MOB target site duplication.
